# Dropsical Affections Successfully Treated with Sugar

**Published:** 1846-02

**Authors:** 


*Dropsical Affections successfully treatrd with Sugar.*—We find in the Gazette Medicale (11th Oct., 1845,) the notice of a collection of cases recently published by M. Bagot, illustrating the beneficial effects of sugar in dropsical affections. It seems to have been first used by Dr. Garnier, who, towards the close of the last century, became affected in the French West Indies, with ascites, for which he was tapped three times, when, looking upon his case as hopeless, he indulged an inordinate propensity to eat sugar. Finding that it agreed with him, he soon made it his sole food, and, to his great astonishment, found his disease rapidly giving way. He was restored to perfect health, and the case published in Paris.

M. Bagot now reports about twenty cases, including almost every variety of dropsy, even one of ovarian encysted dropsy, in which sugar has been used advantageously. The brown sugar is thought preferable to the refined, and should be used almost to the exclusion of all other nourishment—the quantity being increased from a few ounces to a pound per day. The remedy is simple, and certainly worthy of trial.—*Southern Medical and Surgical Journal.*

